# The rapid and efficient strategy for SARS-CoV-2 Omicron transmission control: analysis of outbreaks at the city level

**DOI:** 10.1186/s40249-022-01043-2

**Published:** 2022-11-24

**Authors:** Jin-Xin Zheng, Shan Lv, Li-Guang Tian, Zhao-Yu Guo, Pei-Yong Zheng, Yue-Lai Chen, Shi-Yang Guan, Wei-Ming Wang, Shun-Xian Zhang

**Affiliations:** 1 Department of Nephrology, Ruijin Hospital, Institute of Nephrology, Shanghai Jiao Tong University School of Medicine, Shanghai, 200025 People’s Republic of China; 2 Chinese Center for Disease Control and Prevention (Chinese Center for Tropical Diseases Research), NHC Key Laboratory of Parasite and Vector Biology, WHO Collaborating Centre for Tropical Diseases, National Center for International Research On Tropical Diseases, National Institute of Parasitic Diseases, Shanghai, 200025 People’s Republic of China; 3 Longhua Hospital, Shanghai University of Traditional Chinese Medicine, Shanghai, 200032 People’s Republic of China; 4 Department of Epidemiology and Biostatistics, School of Public Health, Anhui Medical University, Hefei, 230032 People’s Republic of China; 5 School of Global Health, Chinese Center for Tropical Diseases Research, Shanghai Jiao Tong University School of Medicine, Shanghai, 200025 People’s Republic of China; 6 One Health Center, Shanghai Jiao Tong University–The University of Edinburgh, Shanghai, 200025 People’s Republic of China

**Keywords:** COVID-19, SARS-CoV-2, Outbreak, Population flow, Time-varying reproduction number

## Abstract

**Background:**

Severe acute respiratory syndrome coronavirus 2 (SARS-CoV-2) Omicron (B.1.1.529) variant is highly transmissible with potential immune escape. Hence, control measures are continuously being optimized to guard against large-scale coronavirus disease 2019 (COVID-19) outbreaks. This study aimed to explore the relationship between the intensity of control measures in response to different SARS-CoV-2 variants and the degree of outbreak control at city level.

**Methods:**

A retrospective study was conducted in 49 cities with COVID-19 outbreaks between January 2020 and June 2022. Epidemiological data on COVID-19 were extracted from the National Health Commission, People’s Republic of China, and the population flow data were sourced from the Baidu migration data provided by the Baidu platform. Outbreak control was quantified by calculating the degree of infection growth and the time-varying reproduction number (${R}_{t}$). The intensity of the outbreak response was quantified by calculating the reduction in population mobility during the outbreak period. Correlation and regression analyses of the intensity of the control measures and the degree of outbreak control for the Omicron variant and non-Omicron mutants were conducted, respectively.

**Results:**

Overall, 65 outbreaks occurred in 49 cities in China from January 2020 to June 2022. Of them, 66.2% were Omicron outbreaks and 33.8% were non-Omicron outbreaks. The intensity of the control measures was positively correlated with the degree of outbreak control (*r* = 0.351, *P* = 0.03). The degree of reduction in population mobility was negatively correlated with the *R*_*t*_ value (*r* = − 0.612, *P* < 0.01). Therefore, under the same control measure intensity, the number of new daily Omicron infections was 6.04 times higher than those attributed to non-Omicron variants, and the *R*_*t*_ value of Omicron outbreaks was 2.6 times higher than that of non-Omicron variants. In addition, the duration of non-Omicron variant outbreaks was shorter than that of the outbreaks caused by the Omicron variant (23.0 ± 10.7, 32.9 ± 16.3, *t* = 2.243, *P* = 0.031).

**Conclusions:**

Greater intensity of control measures was associated with more effective outbreak control. Thus, in response to the Omicron variant, the management to restrict population movement should be used to control its spread quickly, especially in the case of community transmission occurs widely. Faster than is needed for non-Omicron variants, and decisive control measures should be imposed and dynamically adjusted in accordance with the evolving epidemic situation.

**Supplementary Information:**

The online version contains supplementary material available at 10.1186/s40249-022-01043-2.

## Background

Infectious diseases tend to have unique features that are different from other diseases, and the most important characteristic is their unpredictability and the associated potentially explosive implications [1, 2]. Coronavirus disease 2019 (COVID-19) caused by severe acute respiratory syndrome coronavirus 2 (SARS-CoV-2) and is transmitted by respiratory droplets and contacts, it has posed an enormous threat to public health around the world [3, 4].

COVID-19 endangered human life and health and significantly impacted socio-economic development [1, 5]. SARS-CoV-2 has continuously evolved into novel variants, including Alpha (B.1.1.7), Beta (B.1.351), Gamma (P.1), Delta (B.1.617.2), and Omicron (B.1.1.529), and continuous mutation is expected [6, 7]. For the Delta variant, the basic reproduction number (*R*_0_) is close to 4, the median intergenerational interval is 3 days, and the incubation period is 4.4 days [8]. However, the *R*_0_ of the Omicron variant is close to 10 days, with an even shorter intergenerational interval [9]. The biological properties of the Omicron variant confer it with strong infectivity and rapid and surreptitious transmission, many infections are asymptomatic, and the virus is more likely to escape the immune response generated by previous infections or vaccines [10, 11]. The Omicron variant has a significantly higher breakthrough infection rate than that of the Delta variant, thereby greatly increasing the difficulty in implementing effective control measures [11]. The number of hospitalizations and deaths caused by the Omicron variant has been higher than that caused by the Delta variant and has seriously impacted healthcare systems [12, 13]. Therefore, effective prevention and control measures must be comprehensively applied to interrupt Omicron transmission.

The control measures for the COVID-19 outbreak were based on the biological characteristics of SARS-CoV-2 and intended for long-term application [2, 14]. Previous expertise promoted the idea that herd immunity could be achieved as long as the vaccine coverage rate reached a critical value of over 80% [15, 16]. Thus, even if there were new COVID-19 infections, outbreaks and large-scale epidemics would not occur. However, the Omicron variant emerged and, with it, many more infections, indicating that the current vaccine was not able to prevent infections with the Omicron variant [2, 17]. Specific drugs for SARS-CoV-2 are being researched and developed [2, 18]. The emergence of new mutant variants, the imbalance of global vaccine distribution, and breakthrough infections in vaccinated populations have brought continuous challenges to the effectiveness of vaccinations, resulting in the inability to form a population-wide persistent immune barrier in the short-term [19, 20]. Non-drug interventions such as social distancing, mask-wearing, cleaning hands, and avoiding crowds remain the main prevention and control measures [14, 21]. Studies found that social distancing, including the cancellation of small gatherings and the closure of educational institutions, as well as travel and border restrictions, had the greatest impact on preventing and controlling COVID-19 outbreaks of all existing non-drug interventions [2, 18, 22]. In contrast, the least effective interventions were government-provided and international assistance actions, case tracking, and environmental disinfection and sterilization [18]. When the pandemic broke out on a large scale, implementing stricter measures effectively curbed the COVID-19 pandemic caused by the Omicron variant within a relatively short period and achieved the social management goal of dynamic zero-COVID-19.

At the beginning of 2020, COVID-19 began to spread around the world [23, 24]. In the process of coping with the outbreak, beneficial knowledge was constantly accumulated from relevant experiences. The success of the response to COVID-19 threats came not just from the scientific recognition of the biological characteristics of SARS-CoV-2 variants but also from broad approaches [6, 25]. These approaches included the constant surveillance of SARS-CoV-2 variants, symptom-based surveillance, case isolation, the tracing of close contacts (requiring quarantine in separate facilities) and the contacts of contacts, occupation-based screening, the targeted screening of individuals at high risk of infection, the application of big data in epidemiological investigations, and a set of social distancing measures that included travel restrictions, stringent border control policies, and community confinement, all of which played a complementary role in fighting COVID-19 transmission [6, 25]. However, the Omicron variant began to spread in China in February 2022, and the effective prevention measures based on previous strategies were unsuccessful in preventing outbreaks promptly [26, 27]. Therefore, this study was conducted to explore the relationship between control measures and the transmission process of COVID-19 caused by Omicron and non-Omicron mutants across different cities in China. First, the intensity of the prevention measures and the degree of outbreak control were quantified. Second, the correlation between the intensity of the restriction measures and the degree of outbreak control caused by the Omicron variant and non-Omicron variants was analyzed. The conclusion from the study will provide a reference for formulating better control measures and strategies to effectively confront future COVID-19 outbreaks caused by new SARS-COV-2 variants with high infectivity.

## Methods

### Data source and collection

**Fig. 1:**
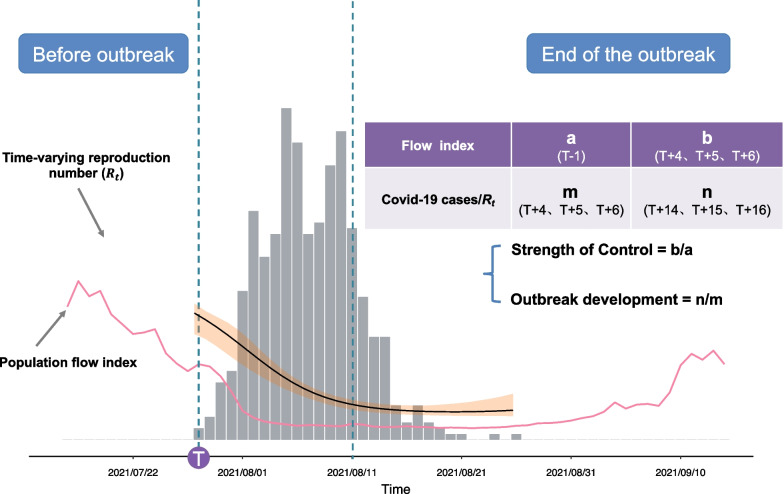
Flow chart demonstrating the quantification of the strength of outbreak control and intensity of control measures

Epidemiological data on COVID-19 were extracted from the National Health Commission, People’s Republic of China (http://www.nhc.gov.cn). The data included outbreak location (provincial level and city level), symptomatic status (including the clinical outcomes of initially asymptomatic infections), date and counts of official reporting (daily asymptomatic infected individuals, daily new cases, and daily death report cases), and other information between January 1, 2020, and June 30, 2022 (Fig. 1). Infectious individual (including asymptomatic person) were all considered to be counted cases in the study.

The population flow data were sourced from the Baidu migration data provided by the Baidu platform (http://www.qianxi.baidu.com). The Baidu Migration Index indicates the scale of population flow between cities and covers 368 cities in China. Two types of population movement data (inflow intensity and outflow intensity) were extracted from the Baidu migration platform between January 1, 2020, and June 30, 2022.

### Study scenarios

Since the large-scale outbreak in Wuhan, China, SARS-CoV-2 has continuously mutated, with concomitant changes in infectivity. Many cities in China experienced outbreaks caused by various SARS-CoV-2 variants (Delta, Omicron, and other major variants). However, the inclusion criteria need to be made to select cities for this study.

The *R*_0_ value describes the potential transmission capacity of a pathogen. The *R*_0_ value of the Delta variant is between 3.2 and 8.0, and the *R*_0_ of the Omicron variant is 3.2 times that of Delta, with a doubling time of approximately 3 days [28], and 1–2 generations of SARS-CoV-2 in one city can cause an outbreak. Hence, we focused on the cities with more than 10 infected persons per day in an outbreak period. Cities with more than 10 new infected individuals daily for 3 consecutive days in normal social environments were included. In contrast, cities were excluded if a COVID-19 outbreak lasted fewer than 7 days or occurred on intermittent days. Cities with one to ten reported infected individuals with community transmission that covered a wide range of time and lasted for 3 months were also excluded, as were cities missing Baidu Migration Index data when the COVID-19 outbreak occurred.

### COVID-19 outbreak definition

The Chinese Center for Disease Control and Prevention (China CDC) defines an outbreak as more occurrences of a particular infectious disease at a specific location and time than expected. However, the definition of a COVID-19 outbreak is usually local-context-related. In non-residential settings, the outbreak criteria were two or more test-confirmed COVID-19 infections among individuals associated with a specific non-residential setting with illness onset dates within 14 days.

COVID-19 outbreak was expressed by the duration of the epidemic. If infected persons were reported in one city at time point T and the city had no infection individuals within 5 days from T-5 to T-1, point T was defined as the time of the onset of the outbreak (Fig. 1). Some cities may have had multiple outbreaks. If the time interval between the end of the first outbreak and the start of the second outbreak was greater than 14 days, it was considered to be a second outbreak.

The time-varying reproduction number (${R}_{t}$) is an important index used to measure SARS-CoV-2 transmissibility during COVID-19 outbreaks. Estimating the increase or decrease in the rate using ${R}_{t}$ is critical for monitoring and adjusting outbreak control measures in the real world. One of the most common methods to estimate ${R}_{t}$ in real-time is through the renewal Eq. (1). This model assumes that the incidence of newly infected individuals on day *t* (*I*_*t*_) can be represented by the following equation [29]:

1 $${I}_{t}\sim \left({R}_{t}{\sum }_{s=1}^{t}{I}_{t-s}{w}_{s}\right)$$

where ${I}_{t}$ is the number of infections that are incident on day *t*, and ${w}_{s}$ is the serial interval distribution. In the renewal equation, the incidence at time *t* (${I}_{t}$) is expressed as a function of the serial interval distribution (${w}_{s}$), the time-varying reproduction number (${R}_{t}$), and the past incidence (${I}_{t-s}$).

The ${R}_{t}$ of the model was quantified using the EpiNow2 package (https://cran.r-project.org/web/packages/EpiNow2) [30]. It estimates the time-varying reproduction number of infectors through the date of infection. The method uses the generation time, and the incidence is computed backward using distributions for the incubation period and reporting delays. In this study, for the non-Omicron variants, a gamma-distributed generation time with a mean of 3.6 days was used in the calculations [31]. For the incubation period, a log-normal incubation period with a mean of 5.2 days was fitted [32]. For the Omicron variant, we referenced the published estimated generation time of 2.7 days for the COVID-19 Omicron wave in Hong Kong [33]. The incubation period of the Omicron variant is set with 3.4 days [34]*.* Ultimately, ${R}_{t}$ was estimated using the EpiNow2 in R software (version 4.2.1, http://www.r-project.org).

### Quantification of outbreak control intensity

Intervention measures were taken to control COVID-19 outbreaks. Thus, the intensity of the outbreak control was quantified based on the ratio of new cases to the extent of change in the *R*_*t*_ value. In the study, for an outbreak event in a specific city, the intensity of the outbreak control was defined as the ratio of the number of new cases on day T + 14, day T + 15, and day T + 16 to the number of new cases on day T + 4, day T + 5, and day T + 6 (Fig. 1), it was shown in formula (2). If the number of new infected persons each day after 2 weeks was less than the numbers of cases on days 4–6 of the outbreak, the outbreak was considered to be controlled. The lower the ratio, the more effective the outbreak control measures.

2 $$Cases \;Control=\mathrm{log}\left(\frac{n}{m}\right)=\mathrm{log}\left(\frac{{Case}_{T14}+ {Case}_{T15}+{Case}_{T16}}{{Case}_{T4}+ {Case}_{T5}+{Case}_{T6}}\right)$$

An *R*_*t*_ of less than 1 suggests that the outbreak was controlled effectively. Therefore, the intensity of outbreak control was defined as the average of the *R*_*t*_ values on day T + 14, day T + 15, and day T + 16 in an outbreak event, as shown formula (3).

3 $${R}_{t} Control=\frac{{R}_{t14}+{R}_{t15}+{R}_{t16}}{3}$$

### Quantification of control measure intensity

The intensity of the prevention and control measures was calculated based on the daily population flow. Baidu Migration Index values, which include inflow and outflow intensity, were used to comprehensively reflect the impact of control measures on population flow. The daily population flow intensity was the average of the inflow intensity and outflow intensity each day. Finally, the intensity of the restraining measures was defined as the ratio of the average population flow on day T + 4, day T + 5, and day T + 6 to that on day T − 1 (Fig. 1), shown in formula (4). Log transformation was additionally applied in the study.

4 $$Strength \;Control = {\text{log}}\left( \frac{b}{a} \right) = {\text{log}}\left( {\frac{{(Flow_{T4} + Flow_{T5} + Flow_{T6} )/3}}{{Flow_{T - 1} }}} \right)$$

## Results

### COVID-19 outbreak and SARS-CoV-2 variants in China

A total of 65 outbreaks in 49 cities were collected across China from January 2020 to June 2022 in this study. The Omicron variant emerged in China in January 2022 and caused 66.2% of all COVID-19 outbreaks [43/65, 95% confidence interval (*CI*) 0.54–0.77], while the outbreak events caused by non-Omicron variants accounted for 33.8% (22/65, 95%*CI* 0.24–0.46). More than 24.4% (12/49, 95% *CI* 0.25–0.38) of the cities experienced two or more outbreaks.

### Relationship between infectious case number and population flow

**Fig. 2:**
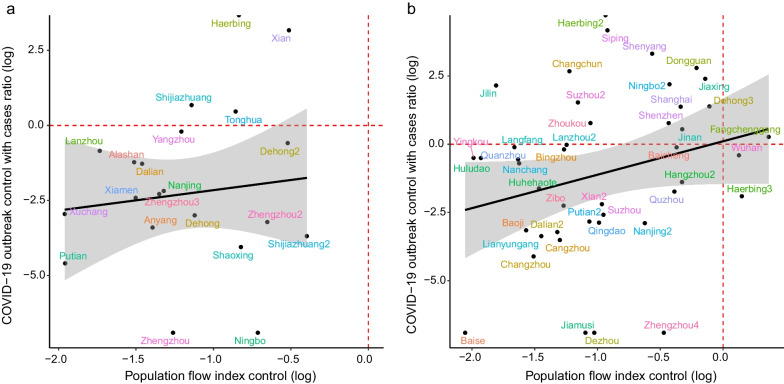
The relationship between intensity of outbreak with infection case control and the degree of population control in Omicron pandemic and non-Omicron pandemic. **a** The scatter plot of infection case control with log transform and population flow index control in non-Omicron pandemic. **b** The scatter plot of infection case control with log transform and population flow index control in Omicron pandemic

**Fig. 3:**
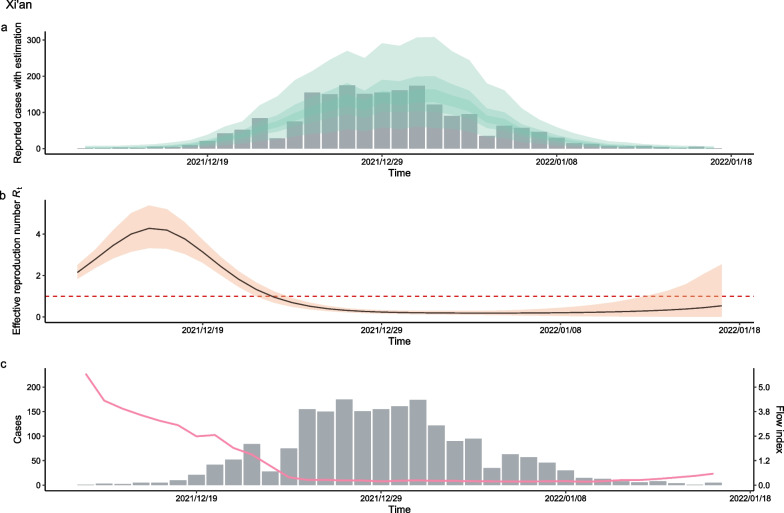
The daily number of reported infection and the population flow index of the non-Omicron outbreak in Xi’an. **a** Simulations of daily cases in by time-varying reproduction number according to daily number of reported infection, the bar chart is the daily number of reported infected individuals. **b** Model estimating the effective reproductive numbers (*R*_*t*_) in each day by EpiNow2 package. **c** The daily number of reported infections (bar plot) and population flow index (pink line)

The relationship between population flow and the increase in cases caused by non-Omicron variants in 22 cities is shown in Fig. 2a, where a positive statistical association was observed (*r* = 0.173, *P* = 0.506). In Fig. 2a, values greater than 0 on the X-axis indicated that control measures were implemented against the pandemic outbreak. The larger the value, the lower the intensity of restraint management. Values less than 0 on the X-axis indicated that more stringent restraint measures were applied to address the outbreak. The smaller the value, the more rapid the reaction time to the COVID-19 outbreak, and the stricter the control measures. Larger values on the Y-axis indicate ineffective control of the COVID-19 outbreak. In contrast, smaller values on the Y-axis represent better effects of the measures used for handling one COVID-19 outbreak. The results showed that the cities where the outbreak was not effectively controlled included Shijiazhuang, Tonghua, Harbin, and Xi’an (Fig. 3). Cities with other COVID-19 outbreak events and dynamic changes in population flow are shown in the Additional file 1. Figure 2a shows that 81.8% (17/22, 95%*CI* 0.565–0.899) of the outbreaks were effectively controlled in China (Additional file 1), suggesting that cities had fewer newly cases after 2 weeks compared to the first 5 days. The results demonstrated that when the population flow decreased by 1%, the number of cases decreased by 2.06 (95%*CI* 0.25–16.54. Table 1).

**Table 1:** Regression model of cases number, *R*_*t*_ value, and intensity of population flow

COVID-19 outbreak control		Intercept	Beta	Exp (Beta) (95% *CI*)	*P* value
Case control*	Non-Omicron	− 0.76	0.72	2.06 (0.25–16.54)	0.13
	Omicron	1.04	1.42	4.16 (1.14–15.18)	0.03
*R*_t_*	Non-Omicron	0.52	− 0.52	–	0.06
	Omicron	1.38	− 1.34	–	< 0.01

Case control means the log of COVID-19 outbreak control with cases ratio. *R*_*t*_ means the COVID-19 outbreak was controlled after 14 days. The “–” symbol indicates the data can not be calculated

The relationship between population flow and increases in the number of infections in COVID-19 outbreaks caused by the Omicron variant is shown in Fig. 2b. A positive statistical correlation was found (*r* = 0.351, *P* = 0.03), and 48.8% (21/43, 95%*CI* 0.346–0.653) of the urban outbreaks were found not to be effectively controlled timely, as reflected in cases numbers that continued to increase within 14 days after the COVID-19 outbreak caused by the Omicron variant. The cities with lower-intensity restrictions included Harbin and Wuhan (Additional file 1). The model showed that the number of infections decreased by 4.16 (95% *CI* 1.14–15.18. Table 1) when population flow increased by 1%.

### Association between COVID-19 transmission and population flow

**Fig. 4:**
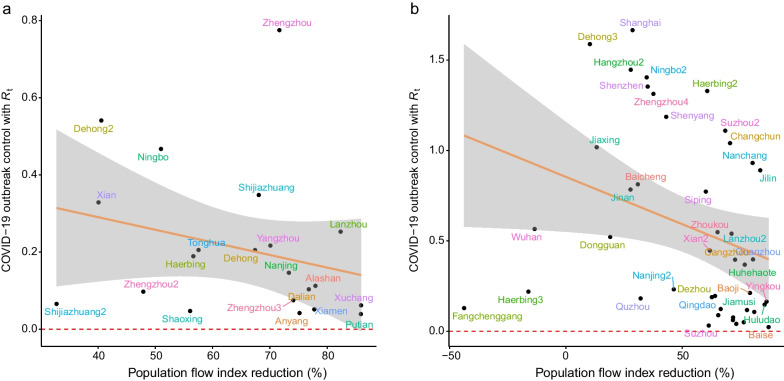
The direct relationship between intensity of outbreak with time-varying reproduction number (*R*_*t*_) and the degree of population control in Omicron pandemic and non-Omicron pandemic. **a** The scatter plot of time-varying reproduction number with population control intensity in non-Omicron pandemic. **b** The scatter plot of time-varying reproduction number with population control intensity in Omicron pandemic

The association between the *R*_*t*_ value for COVID-19 prevalence caused by non-Omicron variants and the degree of reduction in population flow in 21 cities is shown in Fig. 4a. A negative correlation was identified (*r* = − 0.459, *P* = 0.06), where an *R*_*t*_ value of less than 1 indicated better control of the COVID-19 outbreak. Among all cities with outbreaks, those with non-Omicron variants were found to be effectively controlled after 2 weeks. The results showed that the *R*_*t*_ value decreased by 0.52 units when the intensity of restrictive measures increased by one unit (Table 1).

**Fig. 5:**
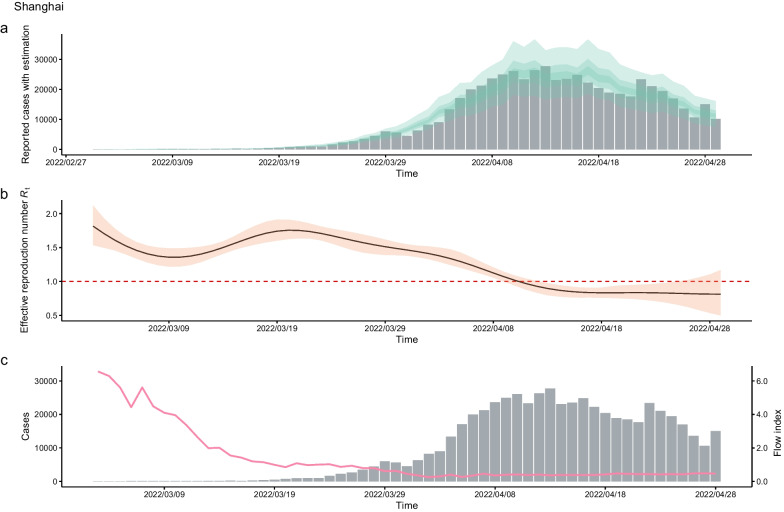
The daily number of reported infections and population flow index of the Omicron outbreak in Shanghai. **a** Simulations of daily infections in by time-varying reproduction number according to daily number of reported infections, the bar chart is the daily number of reported infections. **b** Model estimating the effective reproductive numbers (*R*_*t*_) in each day by EpiNow2 package. **c** The daily number of reported infections (bar plot) and population flow index (pink line)

The relationship between the *R*_*t*_ value of a COVID-19 outbreak caused by the Omicron variant and the degree of reduction in population flow in the 43 cities is shown in Fig. 4b. A negative correlation was observed (*r* = − 0.612, *P* < 0.01). Of the urban outbreaks caused by the Omicron variant, 25.5% (11/43, 95% *CI* 0.149–0.402) were not effectively controlled in short time (Fig. 4b) using the restriction measures previously implemented in response to outbreaks caused by non-Omicron variants. That is, the *R*_*t*_ values remained greater than 1 for 14 days after the outbreak. Shanghai had the highest *R*_*t*_ value (Fig. 5). The results demonstrated that the *R*_*t*_ value decreased by 1.34 units as the intensity of the restriction measures increased by 1 unit.

### Comparison of COVID-19 transmission between Omicron and non-Omicron variants

The relationship between population flow and the growth rate of infection number was statistically significant in COVID-19 transmissions caused by the Omicron variant (*r* = − 0.323, *P* = 0.032), a negative correlation result was found in COVID-19 outbreaks caused by non-Omicron variants (*r* = − 0.341, *P* = 0.129). However, the intercept of the two regression models was different (Table 1). Under control measures of the same intensity, the log-transformation of increases in cases during the Omicron outbreak was 1.80 times higher than that of the non-Omicron variants (Table 1). These results showed that the number of new daily cases caused by the Omicron variant after 2 weeks was 6.04 times higher than the number of patients with non-Omicron variants in situations with the same initial outbreak conditions and the same intensity of restriction measures. In addition, the *R*_*t*_ value of COVID-19 outbreaks caused by the Omicron variant increased by 0.86 compared to COVID-19 outbreaks caused by non-Omicron variants under restriction measures of the same strength. The *R*_*t*_ value of the Omicron variant was 2.6 times higher than that of the non-Omicron variants (1.38/0.52).

### COVID-19 outbreak duration

COVID-19 outbreaks caused by non-Omicron variants lasted an average of 23.0 days [standard deviation (*SD*) = 10.7], whereas Omicron outbreaks lasted an average of 32.9 days (*SD* = 16.3). In the presence of intervention and control measures, the mean duration of COVID-19 outbreaks caused by the Omicron variant was longer than that of outbreaks of non-Omicron variants (*t* = 2.243, *P* = 0.031).

## Discussion

The study showed that if control measures were adopted at an early stage, the explosive growth of infection numbers rarely occurred in non-Omicron outbreaks even if the number of newly infected persons continued to rise. However, the same intensity of restriction measures provided invalid control for Omicron outbreak, and the potential risk of exponential growth of infected individuals and community transmission still existence. The findings from the study can help to render our response to COVID-19 more scientific-based and targeted measures, more targeted approaches must be adopted when it comes to quarantining and transporting patients and close contacts, conducting nucleic acid testing, managing personnel flows, administering vaccination, etc. Optimization of COVID-19 rules based on the nature of SARS-CoV-2 variant can help us to effective response to COVID-19 outbreak, specific measures include shortening quarantine periods for incoming travelers and close contacts of people with COVID-19 infection, cancel the circuit breaker for inbound flights, no longer determine secondary close contacts of confirmed cases, adjust the categories of COVID-19 risk areas to high and low, provide guidance for psychological counseling when necessary, modern material supply must be answered and handled properly. It is necessary to adjust to epidemic situation of infectious diseases and promote the construction of control capacity to coordinate anti-virus policies with social and economic development. It was pointed out that all individuals should adapt to the strong transmissibility of the virus, effectively implement the requirements of early detection, reporting, quarantine and treatment, and adopt swift measures, so as to prevent further expansion of infections or a prolonged prevention and control endeavor.

The Omicron variant became the main pandemic strain worldwide at the end of 2021. There are no specific treatment drugs and preventive vaccine available. Although the fatality rate of Omicron is lower than that of the original strain, the absolute number of deaths in a certain population where the virus is left to spread unchecked would still be very high due to the virus's fast transmission and overextended medical systems. The conclusion of the study suggested that restriction measures reduce transmission and unknown contacts that can be difficult to trace, and the response stresses rapid and targeted action to bring new infections under control in regions with large elderly populations and limited healthcare resources, and it has enabled the country to keep infection and fatality rates very low. Also, similar to our findings, the scientists showed similar approach has been successfully adopted to rapidly subdue several waves of COVID-19 caused with Omicron BA.2 in China [35, 36]. In addition, a large number of established fact proved that effective measures are not taken to respond to Omicron, and the the epidemic will not stop even if the vaccination rate is high [37–39], it can cause massive infections, severe infections and many deaths, especially in a country with a large elderly population and a large number of people with chronic illnesses who are at high risk of severe morbidity and mortality [37–39]. Hence, adherence to positive control measures can also prevent most people from becoming infected and experiencing long-term COVID-19 symptoms (such as fatigue, breathing difficulties, and cognitive impairment) in the severe patients [40]. Thus, implementing positive control measures (dynamic zero-COVID strategy and restriction measures) can protect vulnerable populations and help to cope with the uncertainties associated with emerging variants and the lingering effects of COVID-19 in future. Although critical health services were exempt from the strict suppression strategy, widespread disruptions to routine and emergency non-COVID care due to transport and curfew barriers for patients and health workers, hospitals turning patients away, and supply chain disruptions that affected medicine access and costs [41]. It should be emphasized that life-saving services must be taken into account in the implementation of lockdown. It is necessary that making special arrangements by designating hospitals to receive COVID-19 risk groups and setting up green channels for their hospital visits.

Blocking continuous community transmission can prevent large-scale epidemics, and a comprehensive strategy can promptly and precisely detect and control new outbreaks to halt the transmission of COVID-19 across communities, prevent large-scale viral flare-ups, and achieve a balance between virus control and socioeconomic development [42, 43]. Detecting and identifying the  early infection is crucial for interrupting epidemic. Nucleic acid testing presents the opportunity to identify the number of infectious individuals in the early stages [43, 44], it was regularly performed for high-risk groups, including patients with COVID-19-related symptoms, the close contacts of someone with confirmed infection, as well as the contacts of close contacts, occupational-risk individuals, and personnel in key institutions [35, 44]. In addition, antigen detection could help to identify infected people and prevent community communication before nucleic acid testing were conducted in a large crowd. If transmission can be found within three generations, then an outbreak can be controlled in a small area through epidemiological investigations and targeted epidemic-control measures, and large-scale city lockdown measures will not be necessary [25]. By the time the outbreak spreads to more than the fifth generation, indicating a relatively large transmission scope, or community transmission has occurred in several independent communities, control measures must be strengthened to contain the outbreak [25].

The COVID-19 pandemic has created an unprecedented global crisis [2]. Many factors must be considered in making decisions to implement or terminate preventative measures [25, 45]. These factors mainly include government willingness, the COVID-19 transmission situation, public acceptance of the policies, public health capacity, and medical treatment resources and capabilities, as well as material security resources and capabilities [25]. During the early COVID-19 outbreak, many countries imposed restrictions on population movement, providing time to reduce the incidence, as well as to develop and apply sustained and robust transmission prevention measures [2, 46]. Strict suppression strategy were implemented to slow the spread of SARS-COV-2, prevent case spillovers, and prevent healthcare systems from being overwhelmed [2, 46]. Some cities and countries implemented either complete or partial lockdown. However, individuals affected by restraint measures may experience a loss of personal freedom and autonomy under lockdown, and lower production efficiency from working at home for a long period may result [47, 48]. Therefore, lockdown strategies and other extreme restrictions cannot be sustained for an extended period in one outbreak event.

Governments around the world are now faced with the problems of when and how restrictions should be eased while balancing various health, social, and economic concerns, in certain circumstances that these is not specific therapeutic agents or an effective preventive vaccine, significant weakening of the pathogenicity of the COVID-19 virus has not been observed [2, 46]. The World Health Organization (WHO) warned that lifting lockdown restrictions may trigger a COVID-19 resurgence, but prolonged lockdown may lead to economic collapse in long term [2, 46]. Some studies reported the short-term and long-term effects of COVID-19 control strategies of different intensities on economic development from the perspective of supply chains [5, 36]. The losses incurred by the supply chain from strong restrictive measures were largely dependent upon the duration (primary significance) and severity (secondary significance) of the lockdown and other suppression strategies [5, 36]. However, longer-term lockdown measures were found to be less costly than short-term measures but high-frequency lockdown, and earlier, stricter, and shorter lockdown periods could minimize the overall losses. No matter what control measures are undertaken, the losses to the complex global supply chain will exceed the direct losses caused by the COVID-19 pandemic [5, 36]. Therefore, implementing a series of comprehensive measures that combine short-term intense restraint measures and other effective methods, such tenaciously pursue the general policy of "dynamic zero-COVID.", can coordinate COVID-19 prevention efforts with economic and social development, protect the people's safety and health to the utmost, so as to achieve safe development.

Emerging pathogens with high transmissibility have the characteristic of biological invasion, where the pathogen can rapidly replicate and spread under the synergism of biological features and socioeconomic factors [25, 49]. An intelligent early warning platform for infectious diseases based on multi-point trigger mechanisms and multi-channel surveillance mechanisms can improve the monitoring capacity and control the outbreak of infectious diseases at the early stages [43], the optimised surveillance system have capable of providing early and robust data on a new pathogen. Infectious diseases caused by highly transmissible pathogens can be effectively intercepted by fast and accurate response actions by crossdisciplinary approach and multidisciplinary, multisectoral, and multiprofessional collaboration [50]. Once a highly transmissible pathogen colonizes a very small geographic region, and sporadic infections begin to appear across different regions in one city, a large-scale disease outbreak will soon follow [49]. In this case, precise and differentiated epidemic control strategies based on big data to quickly conduct epidemiological investigations, identify transmission chains, and trace close contacts have modest helpful for quickly controlling COVID-19 outbreaks. Delayed or inadequately prepared control measures are unable to successfully deal with biosecurity threats, resulting in devastating damages and costs. As long as the number of infected individuals increases rapidly, a great burden will be imposed on medical resources, causing medical limiting availability, and threatening the health of patients with underlying diseases, including elderly people, children, and pregnant women. At this stage, large-scale and powerful interventions need to be actively implemented at the beginning of community transmissions during an outbreak as they can successfully deal with highly transmissible infectious diseases within a short period. In addition, the COVID-19 crisis has exposed major weaknesses in health system and social management system [45, 51], a change in health-care framework is needed to improve pandemic prevention [51, 52]. Hence, One Health approach, it has been suggested to address complex global health problems at the human–animal–environment interface, coupled with inter- and trans-disciplinary involvement, that makes broader contributions to achieve optimal public health outcomes by monitoring and managing the interactions between humans, animals, and their environment, it can provide service for formulating policies to promote the prevention and control of emerging infectious diseases [45, 51].

There were several limitations to the study. Firstly, we used the daily number of new cases for each outbreak, and the actual infection time was earlier than the reported time, which may have led to some bias in calculating the effective reproductive number. Secondly, when estimating the *R*_*t*_ for each outbreak, we utilized the parameter for the distribution of delays between symptom onset in a primary and secondary case, and it was different between Omicron and non-Omicron variants. Hence, this may have affected the *R*_*t*_ results in the models. Thirdly, the strength of the governmental control policies was estimated using the Baidu Migration Index. However, mask-wearing, vaccinating, and the closing of public facilities in parks and schools were not considered in the study, which could have decreased the estimates of the strength of the control policies. Finally, real-world effectiveness of strict strategy was obtained from the perspective of preventing infection, it may not be appropriate from a full range of perspectives. Hence, information on outbreak and control policies will be collected systematically to conduct deep analyses.

## Conclusions

The COVID-19 transmission are complex and the intensity of the control measures are varied with different cities. However, the intensity of the control measures can be reflected by population movements. Our findings highlight the decrease of population movement can reduce COVID-19 transmission in cities level, and in response to the Omicron variant or other variants with high infectivity in future, early population movement restriction should be undertaken to control its spread quickly, and decisive control measures should be imposed and dynamically adjusted in accordance with the epidemic situation.

## Supplementary Information

**Additional file 1.** The daily number of reported infections, population flow index and the estimated effective reproductive numbers of SARS-CoV-2 non-Omicron and Omicron outbreak in city levels.

## Data Availability

The data will be available upon requested to first author.

## Abbreviations

CDC: Centers for Disease Control and Prevention (CDC)
*CI*: Confidence interval
COVID-19: Coronavirus disease 2019
SD: Standard deviation
*R*_0_: Basic reproduction number
*R*_t_: Time-varying reproduction number
SARS-COV-2: Severe acute respiratory syndrome coronavirus 2
WHO: World Health Organization
