# Supplementary material for: The rapid and efficient strategy for SARS-CoV-2 Omicron transmission control: analysis of outbreaks at the city level
Source: Infect Dis Poverty. 2022 Nov 24;11:114. doi: 10.1186/s40249-022-01043-2 (PMC9694873; doi:10.1186/s40249-022-01043-2)
Supplement: Supplementary file 1 — Additional file 1. The daily number of reported infections, population flow index and the estimated effective reproductive numbers of SARS-CoV-2 non-Omicron and Omicron outbreak in city levels. [file 40249_2022_1043_MOESM1_ESM.docx]

**Additional File**

**Additional materials**

Study included multi-outbreaks of COVID-19 in 49 cities, we modeled the daily number of reported cases with time-varying reproduction number in each outbreak. We also collected the population flow index from Baidu Migration Index in each cites.

So, in this additional materials, we visualized real daily reported and population flow index in each outbreak by cities names and the outbreak time.

**Content**

Non-Omicron outbreak cities

Omicron outbreak cities

**Figures:**

Non-Omicron outbreak cities

(A) The daily number of reported cases (bar plot) and population flow index (pink line). (B) Model estimating the effective reproductive numbers (*R_t_*) in each day by EpiNow2 package.

**Figures:**

Omicron outbreak cities

(A) The daily number of reported cases (bar plot) and population flow index (pink line). (B) Model estimating the effective reproductive numbers (*R_t_*) in each day by EpiNow2 package.
